# Integrating quantitative biometrics and automated risk stratification for ophthalmic surgical triage

**DOI:** 10.3389/fmed.2026.1975712

**Published:** 2026-10-30

**Authors:** Jacob Craft, David Fleischman

**Affiliations:** Ophthalmology, UNC School of Medicine, Chapel Hill, NC, United States

**Keywords:** cataract surgery outcomes, clinical decision support, electronic health record, perioperative risk stratification, social determinants of health, surgical cancellation prediction

## Abstract

Ophthalmology generates extensive quantitative data from ocular biometry and imaging, yet these device-derived measurements are often separated from medical, social, and utilization data in the electronic health record (EHR). No validated ophthalmic tool currently integrates these streams for real-time, patient-specific perioperative decision support. This mini-review examines how automated EHR-based risk stratification and quantitative ocular biometrics could support perioperative decision-making. Across surgical specialties, cancellation models using demographic, comorbidity, socioeconomic, and appointment-related variables have achieved development areas under the curve (AUC) of 0.75–0.92 with a validation range of 0.70–0.74. When the bundle is paired with targeted care pathways, reduced same-day cancellations have been observed, although bundle designs prevent quantifying the independent contribution of the model. In cataract surgery, Epic's Health Composite Score independently predicts same-day cancellation but provides only limited discrimination, and does not predict operative or postoperative outcomes, which remain more closely associated with ocular complexity and surgeon-related factors. Cataract and glaucoma literature identifies additional candidate predictors of technical complexity, surgical outcomes, and access, including pupil and lens characteristics, axial length, anterior chamber anatomy, prior ocular surgery, angle dimensions, conjunctival imaging, frailty, and social vulnerability. We propose an EHR-integrated framework with endpoint- and procedure-specific models for surgical readiness, technical complexity, and adverse outcomes. Each output should be linked to a defined response, such as patient navigation, anesthesia review, case assignment, operative setting, equipment planning, or intensified postoperative follow-up. Prospective multicenter development should assess whether model-guided interventions improve access, efficiency, and outcomes.

## Introduction

Ophthalmology is distinctive among surgical specialties in the volume and precision of the quantitative data it generates. Routine care produces highly reproducible, procedure-specific ocular measurements, including biometry, keratometry, anterior chamber depth, axial length, and anterior segment imaging, alongside the medical and social information contained in the EHR. Ocular measurements reside in device databases, while medical comorbidity and social information sit within the general EHR. This separation limits the ability of automated tools to reason about the full picture of a surgical patient.

Ophthalmic surgical decisions depend on patient-level and eye-specific risk. A patient may be medically and logistically ready for surgery yet present a technically formidable eye or be medically complex yet require a routine procedure. Consider a healthy patient with pseudoexfoliation and a shallow anterior chamber. This patient faces a complex surgical course, yet a generic composite health score would classify them as low risk.

Surgical cancellation is one important application of this integrated approach, not the sole motivation for it. The burden of cancellation in ophthalmology is nonetheless acute. Cancellation rates approaching 9% have been documented for glaucoma surgery, with mean delays of 63 days per cancelled case (1). Safety-net hospitals have reported baseline cancellation rates exceeding 17%, most occurring on the day of surgery (2, 3). Among 123 glaucoma-practice cancellations, only 28% were judged preventable, 50% were unpreventable, and 23% had no reason recorded. The leading preventable causes were lack of transportation (34%), lack of timely clearance (26%), and insurance-related issues (14%). Any cancellation-prediction system therefore operates against a ceiling of roughly a quarter of events, and the preventable causes are logistical rather than ocular (1).

The financial consequences are substantial, and operating room time is now increasing in value (1, 4). Blanket preoperative testing in ophthalmology has shown no benefit for low-complexity procedures (5). What is needed instead is targeted, procedure-aware risk stratification, and the data to build it already exist within ophthalmic practice.

EHR-derived composite score utilization could bridge this gap by synthesizing patient demographics and healthcare utilization patterns into actionable risk metrics. Across surgical specialties, structured care bundles incorporating purpose-built cancellation prediction models have achieved discriminative performance while generating substantial cost savings (6, 7). In ophthalmology, the Epic Health Composite Score has been independently associated with same-day cataract surgery cancellations (8).

Large ophthalmic data infrastructures such as the IRIS Registry and the National Ophthalmology Database aggregate linked clinical, procedural, and outcome data for benchmarking and research (9–11), but they do not link device-derived quantitative biometrics with patient-level social and utilization data to generate a real-time, patient-specific perioperative triage output. It is integration for that decision-support purpose that no validated ophthalmic tool currently provides.

Relevant literature was identified by searching PubMed for records published 2,000 through 2026, using combinations of the terms surgical cancellation, prediction model, risk stratification, cataract, glaucoma, electronic health record, ocular biometrics, and social determinants of health. Reference lists of included articles were screened for additional sources.

## Automated EHR-based prediction of surgical readiness and cancellation

Automated EHR-based models can identify patients who may need added perioperative support with little or no additional clinician data entry. This advantage applies to the patient-level stream but does not always extend to device-derived ocular measurements due to integration variance between clinical settings. A systematic review of six cancellation prediction models encompassing 759,337 elective surgical procedures found methods ranging from logistic regression to machine learning, with predictors spanning demographic, socioeconomic, comorbidity, surgical, and appointment-related domains (6). Discriminative performance in development datasets ranged from AUC 0.75 to 0.92, while validation datasets yielded more attenuated estimates of AUC 0.70–0.74 (6). On formal appraisal with PROBAST, three of the six models evaluated were noted to have a high risk of bias, with two being low risk and one unclear which was also responsible for the upper AUC bound of 0.92. Of note, the reviewed models showed only moderate discrimination with significant heterogeneity and limited calibration reporting. Therefore, discriminative performance alone should not be taken as evidence of readiness for deployment.

Prediction is only useful when it is connected to an intervention. The clearest demonstration comes from Wongtangman et al., who developed and validated a 29-predictor cancellation score (AUC 0.79 development, 0.73 on temporal validation in a later cohort drawn from the same university-affiliated hospital network). When embedded within a structured care bundle, it combined automated screening with patient engagement tools (7, 12). Following implementation in an ambulatory otorhinolaryngology program, same-day cancellation rates declined from 12.3% to 8.4%, with estimated cost savings of $150,200, a 35.3% reduction in cancellation-related expenditure (7, 12). Given the four component bundle design, the contribution of the risk score cannot be isolated. The reported mechanism points substantially to communication. Patient engagement via portal or text rose from 75.9% to 90.8% with a corresponding fall in day-of-surgery missed appointments from 7.2% to 4.5%, being the largest single driver of improvement. The bundle therefore reduced cancellations and costs, but its design cannot attribute that effect to the model itself.

Suarez et al. developed the first cancellation prediction model to integrate comprehensive social determinants of health data, achieving an AUC of 0.80 at a safety-net hospital with predictors including wheelchair use and other markers of social vulnerability (3). These findings suggest that multidomain models incorporating SDOH, comorbidity, frailty, and utilization data may add predictive information beyond narrower approaches for cancellation prediction.

Within ophthalmology, the most thoroughly evaluated EHR-derived tool for cancellation prediction is the Epic Health Composite (EHC) Score, a proprietary composite health metric. In our prior retrospective cohort of 1,204 cataract surgery patients, each 5-point increase in the EHC was independently associated with a 23% higher odds of same-day cancellation (adjusted OR 1.23; 95% CI, 1.10 to 1.38; *p* < 0.001), with limited discrimination (AUC 0.632) (8). Of note, an AUC of 0.632 is inadequate for individual-level triage and should be read as evidence that an EHR-derived signal for cancellation exists rather than as evidence of a deployable tool. Critically, the same study found that the EHC showed no association with operative duration, anesthesia selection, surgical complications, prolonged postoperative inflammation, or visual outcomes, which were instead predicted by ophthalmic-specific factors including cataract complexity, prior ocular surgery, and retinal or optic nerve pathology (8). This dissociation is the central limitation of generic scores with ocular and procedural variables being required to anticipate ophthalmic outcomes.

## Quantitative and clinical risk stratification in cataract surgery

Prognostic stratification for cataract surgery is multifactorial. A systematic review of cataract surgery risk stratification models identified eight published models which were designed to predict posterior capsule rupture and zonular complications (13) The recognized high-risk ocular characteristics are well catalogued and include small pupil size, pseudoexfoliation, zonular weakness, axial length extremes, shallow anterior chamber, dense brunescent or white intumescent cataract, posterior polar cataract, and prior ocular surgery or trauma (9, 14). The Royal College of Ophthalmologists’ National Ophthalmology Database, identified pseudoexfoliation or phacodonesis (OR 3.47) and brunescent, white, or mature cataract (OR 2.41) as some of the strongest ocular predictors of PCR, along with shallow anterior chamber depth (9, 10). An anterior chamber depth of less than 2.5 mm may indicate zonular weakness and has been associated with a roughly fivefold increase in complication risk in pseudoexfoliation eyes (15).

The Triemli Cataract Score represents one of the more structured attempts to translate preoperative ocular risk assessment into an operational surgical triage tool. The tool classifies cases as routine, complex, or highly complex to guide resource allocation, surgeon assignment, and perioperative preparation. Following implementation at a Swiss hospital, intraoperative complication rates trended downward, from 9.5% pre-implementation to 7.4% post-implementation (*p* = 0.054), and differed significantly across risk categories (*p* = 0.014), at 5.6%, 11.2%, and 8.8% for routine, complex, and highly complex cases respectively (16). The higher complication rate in the complex group relative to the highly complex group is counterintuitive and may reflect the concentration of advanced resources and personnel on the highest-risk cases. A subsequent study at the same institution found that the TCS did not reach conventional statistical significance as an independent predictor of PCR or vitreous loss in multivariable analysis (OR 1.79, *p* = 0.057), while pseudoexfoliation remained an independent risk factor (OR 2.38, *p* = 0.013) (17). The systematic review found that most published models lacked external validation among other limitations which suggests the TCS requires further study before clinical adoption.

A Taiwan national database study of 2,046 complicated cataract cases identified hypertension (aOR 2.33), diabetes (aOR 2.82), congestive heart failure (aOR 2.89), renal failure requiring hemodialysis (aOR 2.94), rheumatic disease (aOR 1.97), and hyperlipidemia (aOR 1.70) as being independently associated with severe postoperative ophthalmic complications (18). Demographics also influence outcome with the Sight Outcomes Research Collaborative (SOURCE) study, a 16-center analysis of 78,565 eyes, identifying Black race (HR 1.66), male sex (HR 1.14), prior intraocular surgery (HR 1.18), combined intraocular procedures (HR 1.33), surgical complexity (HR 1.23), and poorer preoperative visual acuity (HR 1.10) as independent predictors of any postoperative complication (19). These hazard ratios must be read against the absolute event rate: the overall 6-month complication rate was 4.16%, with severe adverse complications at 0.18% (19). Against a 4.16% base rate, an HR of 1.66 implies a difference in the absolute risk likely large enough to shift care patterns systematically by race if operationalized, but, in an effort to preserve fairness we suggest reserving the use of race for outcome auditing. The cataract literature suggests ocular and surgical factors remain the primary determinants of intraoperative difficulty and the appropriate primary targets of ophthalmic risk stratification, while specific systemic diseases and sociodemographic factors could contribute to other risk through distinct mechanisms.

## Ocular biometrics and patient-level risk in glaucoma surgery

Glaucoma surgery illustrates why ophthalmic prediction must be procedure specific. Unlike cataract surgery, glaucoma encompasses heterogeneous procedures with different mechanisms, postoperative demands, definitions of success, and time horizons for failure. Long-term failure rates in randomized tube-versus-trabeculectomy trials (20) underscore the consequences of procedure selection and postoperative wound healing, but these efficacy endpoints are not directly comparable with intraoperative cataract complications.

Quantitative anterior segment OCT (AS-OCT) can inform preoperative planning with Penzner et al. finding that 37.6% of open-angle eyes had an angle-opening distance at Schwalbe's line too small to safely accommodate standard tube shunt devices (21). Likewise, conjunctival imaging parameters have been associated with filtration surgery outcomes. Mastropasqua et al. demonstrated lower conjunctival stromal thickness inversely correlated with postoperative intraocular pressure (r = −0.408) and independently associated with surgical failure (OR 0.941) (22).

Sugihara et al. found that geriatric assessment measures, including the Mini-Cog, G8, and age-adjusted Charlson Comorbidity Index, improved prediction of severe hemorrhagic complications after glaucoma surgery (23). The reported AUC of 0.96, however, reflects the best-performing algorithm in a single-center retrospective series of 729 eyes from 464 patients with roughly 8% severe hemorrhagic events, without external validation but did adjust for within-patient clustering. In a separate single-center study, Groothoff et al. found that each 0.1-unit increase in the electronic frailty index was associated with a lower likelihood of receiving glaucoma surgery and a longer time to surgery (24). Medicare claims analyses similarly found lower rates of outpatient glaucoma care and surgery among moderately to severely frail beneficiaries (25). These studies suggest that frailty may influence both perioperative risk and access to treatment, but they do not establish whether lower surgical utilization reflects appropriate clinical selection, undertreatment, or both.

Glaucoma surgery also imposes a heavy postoperative follow-up burden, which frail patients may be least able to sustain. Patients residing more than 50 miles from clinic had greater odds of loss to follow-up than those within 25 miles (OR 3.47), and patients lost to follow-up lived a mean of 12.59 miles farther from clinic (26). Residence in areas of higher social vulnerability index has likewise been independently associated with delayed glaucoma surgery (27).

The imaging and biometric studies cited here demonstrate potential but are not validated prediction tools ready for clinical deployment. Collectively, glaucoma surgery literature demonstrates that ocular, systemic, and sociodemographic factors each contributing independent signals.

## An integrated framework

Automated EHR risk stratification and quantitative ocular biometrics address different dimensions of perioperative risk. EHR-derived factors characterize the patient's medical and logistical readiness, while ocular measurements characterize the technical demands and procedure-specific risks of surgery. The value lies in combining them within a shared decision-support framework that produces endpoint-specific outputs, each tied to a defined operational response. The EHR specific data could inform each endpoint where an association exists. For example, systemic comorbidity, healthcare utilization, sex, frailty, diabetes may be used to adjust the surgical risk, and technical complexity (9, 18, 19, 23). The ocular biometric stream can then be incorporated, likely strengthening the tool's predictive value for the surgical risk and complexity endpoints.

The MySurgeryRisk multicenter model identified primary procedure code as the most important predictor of postoperative complications. Clinician specialty, or surgeon identity in the sensitivity analysis, was the second most contributory signal (28). Although specific to inpatient surgery, this finding suggests the principle that risk-stratified triage should match patients to surgical teams whose expertise aligns with case complexity, rather than relying on comorbidity burden alone.

Automated risk scoring is technically feasible at scale within existing EHR environments. An automated machine learning model deployed across 20 hospitals achieved AUROCs of 0.946 for mortality and 0.899 for major cardiovascular events using only preoperative EHR data, refreshed automatically without manual extraction (29). EHR-based clinical decision support has also reduced unnecessary preoperative testing by 12.2% and associated costs by 22% (30)., improving guideline-concordant ordering (31), and enhancing VTE prophylaxis adherence with measurable improvements in clinical outcomes (32).

A modular architecture addresses integration by maintaining a shared EHR-based infrastructure while allowing the relative weighting of input domains to vary by procedure type. (Figure 1) The readiness and cancellation model runs when surgery is requested or scheduled with automatic population of patient-level EHR variables. It would extract high-risk systemic diagnoses, validated social vulnerability indicators, healthcare utilization patterns, and frailty-related markers without additional data entry. The complexity and outcome models, however, depend on the ocular stream, often obtained at or after the decision to operate. Notably, anterior segment OCT is not standard in most cataract or glaucoma workflows, and many glaucoma procedures involve no biometry at all. These models should therefore fire later in the pathway, on completion of biometry or finalization of the relevant examination. In parallel, ocular measurements are imported from device databases with automated or semi-automated entry where device integration is incomplete. The planned procedure then determines which ocular predictions are relevant and how input domains are weighted. Importantly, we suggest that outputs requiring an unavailable input should be suppressed and labelled as “not assessed” to improve adaptability in settings where ocular biometry is not always available. Endpoint-specific risk estimates then surface within the existing EHR workflow so that cancellation, technical complexity, and surgical outcomes become use cases of one framework.

**Figure 1:**
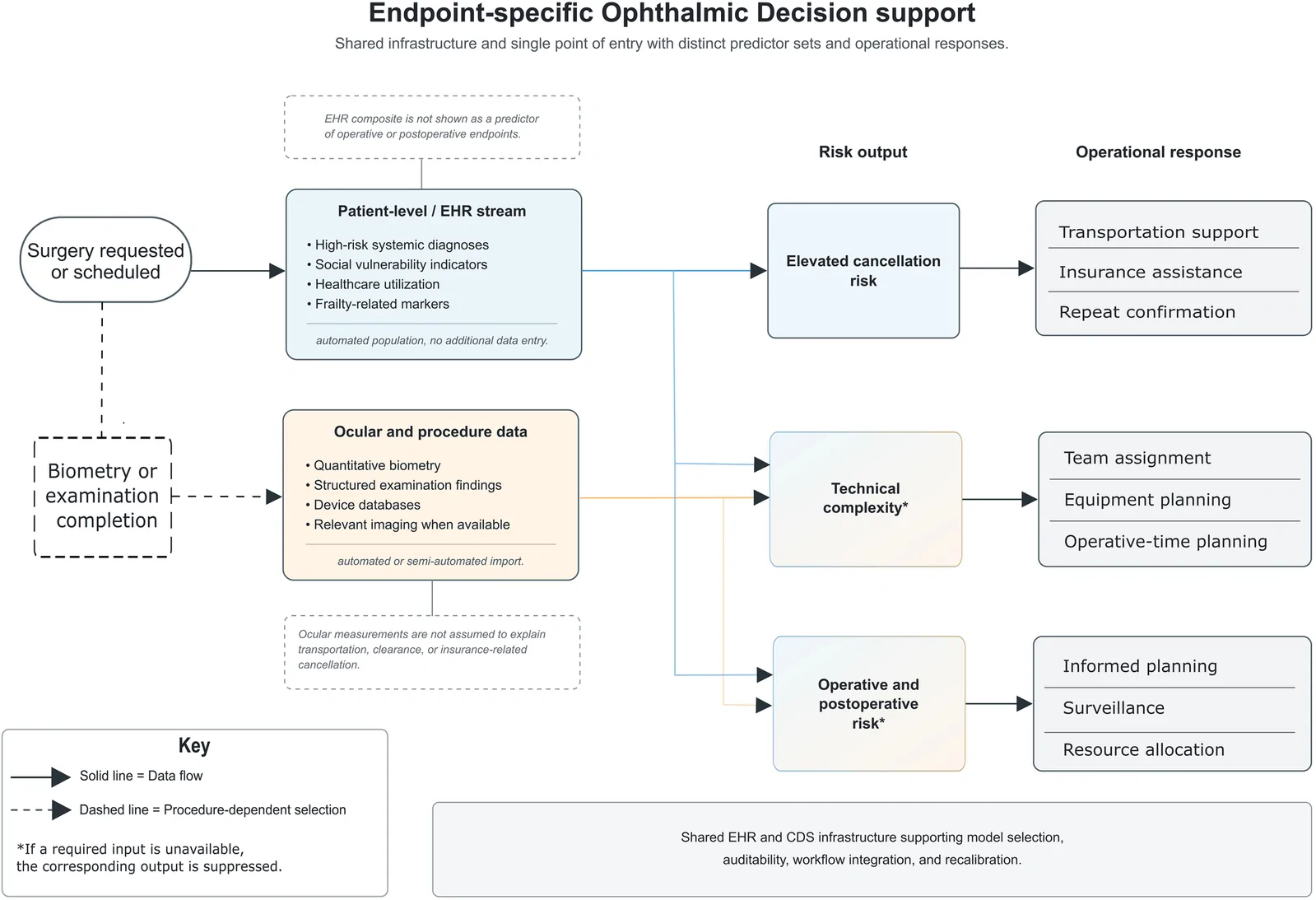
Proposed modular, EHR-integrated framework for endpoint-specific ophthalmic perioperative risk stratification. The readiness and cancellation model runs when surgery is requested or scheduled with automatic population of patient-level EHR variables. The complexity and outcome models depend on the ocular stream. The planned procedure then determines which ocular predictions are relevant and how input domains are weighted. The model outputs separate, endpoint-specific risk estimates, each tied to a defined response.

It is highly likely that model deployment will alter the outcomes they are trained to predict. For example, routing complex cases to senior surgeons changes downstream complication rates, which both degrades discrimination and the potential for retraining on post-deployment data. This pattern is consistent with the lower complication rate observed for highly versus moderately complex cases after Triemli implementation (16). A successful triage tool will appear to lose discrimination and will require post-deployment monitoring and recalibration to account for treatment-paradox confounding.

## Discussion and future directions

The available evidence (Table 1) supports integration through a shared data infrastructure, but not the creation of a single universal ophthalmic risk score. EHR-derived patient factors and quantitative ocular biometrics provide complementary information and should inform endpoint- and procedure-specific models, each linked to a defined clinical response.

**Table 1:** Summary of prediction models and risk stratification tools for surgical cancellation and ophthalmic surgical outcomes.

Study/tool	Specialty/procedure	Study design & sample size	Predictor domains	Primary endpoint	Performance	Validation status
Systematic review (6 models)	Multi-specialty elective surgery	Systematic review; 759,337 procedures	Demographic, socioeconomic, comorbidity, surgical, appointment	Day-of-surgery cancellation	AUC 0.75–0.92 (development); 0.70–0.74 (validation)	PROBAST appraisal—3/6 models with high risk of bias, 2/6 were low risk and 1/6 unclear (also responsible for upper AUC bound of 0.92)
Wongtangman et al. (score)	Elective surgery across specialties	Retrospective development; 305,274 cases	29-predictor multidomain	Day-of-surgery cancellation	AUC 0.79 (developmental) and AUC 0.73 (temporal validation);	Temporal validation
Wongtangman et al. (bundle)	Otorhinolaryngology	Bundle implementation study; 1,548 cases	29-predictor multidomain	Day-of-surgery cancellation	cancellations 12.3%→8.4%; savings $150,200	no bundle validation completed
Suarez et al.	Safety-net hospital, urology	Retrospective single-center cohort; 778 patients with 136 cancellations events	SDOH, demographics, utilization	Day-of-surgery cancellation	AUC 0.80 (95% CI 0.70 to 0.89), kappa 0.20, specificity 55%^a^; key predictors: wheelchair use (OR 3.17), Black race (OR 2.62), incarceration (OR 2.00)	internally cross-validated only
Epic Health Composite Score	Ophthalmology (cataract)	Retrospective cohort; 1,204 patients	EHR-derived composite health burden	Same-day cataract cancellation	AUC 0.632; aOR 1.23 per 5-point increase	Single-center retrospective association study, no external validation
Cataract risk stratification models (systematic review)	Ophthalmology (cataract)	Systematic review; 8 models	Ocular/biometric (lens instability, pupil)	PCR/zonular complications	Limited multivariable modeling	Only three of the eight models externally validated
Triemli Cataract Score (implementation)	Ophthalmology (cataract)	**Pre-post implementation; single center; 1,776 eyes (704 pre, 1,072 post)**	Ocular risk factors (tiered complexity)	Intraoperative complications	Complications 9.5%→7.4% (*p* = 0.054); by tier 5.6%, 11.2%, and 8.8%	no external validation
Triemli Cataract Score (predictor analysis)	Ophthalmology (cataract)	retrospective cohort of 2,719 eyes evaluating a Triemli Cataract Score (TCS) threshold of ≥3	Ocular risk factors (tiered complexity)	PCR or vitreous loss	OR 1.79 for PCR/VL (*p* = 0.057), pseudoexfoliation OR 2.38 (*p* = 0.013)	no external validation
Taiwan national database	Ophthalmology (cataract)	Matched case- control study of 2,046 complicated cases vs. 8,184 controls	Systemic comorbidities	Severe postoperative complications	HTN aOR 2.33; DM aOR 2.82; CHF aOR 2.89; renal failure aOR 2.94	not developed or validated as a prediction model
SOURCE study	Ophthalmology (cataract)	Multicenter (16 centers); 78,565 eyes	Sociodemographic + ocular	Postoperative complications	Black race HR 1.66; male HR 1.14; poor VA HR 1.10; severe: male HR 1.54, poor VA HR 1.62	not validated as a prediction model
Sugihara et al.	Ophthalmology (glaucoma)	Single-center retrospective, 729 eyes from 464 patients, about 8% severe hemorrhagic events	Geriatric/frailty assessment (Mini-Cog, G8, CCI)	Hemorrhagic complications	AUC up to 0.96 for the best-performing algorithm, eye-level analysis which accounted for within-patient clustering	no external validation
MySurgeryRisk	Multi-specialty (inpatient)	Multicenter (14 OneFlorida + institutions)	Procedure code, specialty, EHR data	Prediction of ICU admission, ventilation, AKI, and mortality	Mortality AUROC 0.95	Internal validation

Studies are organized by endpoint (cancellation prediction vs. complication risk stratification) and specialty scope. Predictor domains, study designs, sample sizes, and key performance metrics are presented to facilitate comparison across methodologically heterogeneous studies.

AUC, area under the receiver operating characteristic curve; aOR, adjusted odds ratio; HR, hazard ratio; OR, odds ratio; PCR, posterior capsule rupture; VL, vitreous loss; HTN, hypertension; DM, diabetes mellitus; CHF, congestive heart failure; VA, visual acuity; AUROC, area under the receiver operating characteristic curve; CCI, Charlson Comorbidity Index; SDOH, social determinants of health; EHR, electronic health record.

a dds ratios come from a lasso-penalized model with shrunken coefficients and should not be read as conventional independent effect estimates.

Several limitations warrant consideration. The proposed framework remains conceptual and has not undergone prospective validation. The evidence reviewed is methodologically heterogeneous and includes administrative datasets, single-center cohorts, variable outcome definitions, and limited reporting of calibration and external validation. Evidence maturity is also uneven across endpoints, with the readiness and cancellation arm resting on larger multi-institution data and a real-world deployment while the complexity and outcome arms rest on single-center, externally unvalidated work, so the three should not be read as equally ready for clinical use. Because this is a narrative mini-review, relevant studies may have been missed. Generalizability across community, ambulatory, safety-net, and international settings also remains uncertain.

The proprietary nature of the Epic Health Composite Score, whose component variables and weighting are not publicly disclosed, underscores the importance of transparency in future models. A tool that outputs a risk estimate and drives case assignment and equipment planning has a plausible claim to being a regulated Clinical Decision Support software. Whether such a tool falls within the non-device CDS exemption relies on the treating clinician option to review the basis for each recommendation (33). The framework should therefore favor transparent, explainable models with auditable logic.

Prediction must also be linked to an effective intervention. The Wongtangman et al. care bundle improved appointment adherence through automated screening, messaging, and targeted preoperative processes, but patients whose risk is driven by transportation, language, financial, or insurance barriers may require institution-specific navigation and support. Prospective studies should therefore assess whether risk-stratified decision support improves the appropriateness, safety, and accessibility of surgical care. Frailty and adverse social conditions are also correlated constructs and may provide overlapping information. Models that include both should address redundancy, missingness, calibration, and subgroup performance over treating them as fully independent, additive risk factors (34, 35).

The next priority is the prospective development and validation of a modular family of ophthalmology-specific models. These models should combine transparent EHR-derived variables with quantitative ocular biometrics and procedure-specific clinical findings and should be tested across both routine and complex ophthalmic procedures in diverse practice settings. Evaluation should extend beyond discrimination to include calibration, external validity, workflow burden, and clinical utility. Explicit safeguards are also required so that risk markers, such as race, do not become access barriers. Models should need to demonstrate calibration and comparable error rates across subgroups before use with post-deployment subgroup monitoring. To uphold fairness, we suggest reserving race for outcome auditing as a route to improve surgical equity.

The Wongtangman et al. bundle provides a useful implementation template for pairing prediction with intervention, though its design leaves the model's independent effect unresolved and future studies should be structured to isolate it. By combining automated patient-level risk assessment with quantitative, procedure-specific ocular data, ophthalmology can leverage its uniquely rich measurement environment to provide decision support that neither data stream could deliver alone.

## References

[1] Mehran N Ojalvo I Myers JS Razeghinejad R Lee D Kolomeyer NN. Surgical cancellations in glaucoma practice: causes. Delays, and effect on patient care and revenue. Ophthalmology Glaucoma. (2021) 4(4):427–32. 10.1016/j.ogla.2020.12.006 33338680

[2] Srivastava S Gandhi S Romero Medina S Tewfik G Chaudhury F Narula N. Surgical case cancellations at a safety net hospital: implications for patient care and resource management. World j Surg. (2025) 49(9):2493–500. 10.1002/wjs.70007 40689751

[3] Suarez PA Srirangapatanam S Leng L Momodu MM Neuhaus J Bayne DB. Enhancing surgical efficiency: predicting same-day cancellations in urologic procedures. World J Urol. (2026) 44(1):48. 10.1007/s00345-025-06155-6 PMC12712017 41405624

[4] Ashrafi A Maggard-Gibbons M Childers CP. Costs, charges, and revenue of hospital operating rooms in California. JAMA Surg. (2026) 161:901–7. 10.1001/jamasurg.2026.2355 42384389 PMC13324936

[5] Keay L Lindsley K Tielsch J Katz J Schein O. Routine preoperative medical testing for cataract surgery. Cochrane eyes and vision group, editor. Cochrane Database Syst Rev. (2019) 2019(1):CD007293. 10.1002/14651858.CD007293.pub4 PMC6353242 30616299

[6] Sardesai T Hobbs L Phillips C Ang G Bashford T Kohler K. A systematic review of efforts to predict day of surgery cancellation. Eur J Anaesthesiol. (2026) 43(8):692–702. 10.1097/EJA.0000000000002370 42383309 PMC13336580

[7] Wongtangman K Semczuk P Freda J Smith RV Pushparaj V Beckham D. The effect of a bundle intervention for ambulatory otorhinolaryngology procedures on same-day case cancellation rate and associated costs. Anaesthesia. (2024) 79(6):593–602. 10.1111/anae.16247 38353045

[8] Phan C Amiri I Levi M Fleischman D. Predictive utility of Epic's Health composite score for same-day cancellation in cataract surgery. Clin Ophthalmol. (2026) 20:1–10. 10.2147/OPTH.S601482 PMC13142688 42094810

[9] Sim PY Donachie PHJ Day AC Buchan JC. The royal college of ophthalmologists’ national ophthalmology database study of cataract surgery: report 17, a risk factor model for posterior capsule rupture. Eye. (2024) 38(18):3495–503. 10.1038/s41433-024-03344-2 39294232 PMC11621766

[10] Day AC Norridge CFE Donachie PHJ Barnes B Sparrow JM. Royal college of ophthalmologists’ National Ophthalmology Database Study of Cataract Surgery: report 8, cohort analysis of the relationship between intraoperative complications of cataract surgery and axial length. BMJ Open. (2022) 12(8):e053560. 10.1136/bmjopen-2021-053560 35985773 PMC9396167

[11] Ciociola EC Sekimitsu S Smith S Lorch AC Miller JW Elze T. Racial disparities in glaucoma vision outcomes and eye care utilization: an IRIS registry analysis. Am J Ophthalmol. (2024) 264:194–204. 10.1016/j.ajo.2024.03.022 38548127

[12] Wongtangman K Azimaraghi O Freda J Ganz-Lord F Shamamian P Bastien A. Incidence and predictors of case cancellation within 24 h in patients scheduled for elective surgical procedures. J Clin Anesth. (2022) 83(110987):110987. 10.1016/j.jclinane.2022.110987 36308990

[13] Kang C Zhu AS Waldman O Kashner TM Greenberg PB. Cataract surgery risk stratification models: a systematic review. Graefes Arch Clin Exp Ophthalmol. (2025) 263(5):1229–38. 10.1007/s00417-025-06761-5 39900805

[14] Miller KM Oetting TA Tweeten JP Carter K Lee BS Lin S. Cataract in the adult eye preferred practice pattern®. Ophthalmology. (2022) 129(1):P1–P126. 10.1016/j.ophtha.2021.10.006 34780842

[15] Küchle M Viestenz A Martus P Händel A Jünemann A Naumann GO. Anterior chamber depth and complications during cataract surgery in eyes with pseudoexfoliation syndrome. Am J Ophthalmol. (2000) 129(3):281–5. 10.1016/s0002-9394(99)00365-7 10704540

[16] Kaiser K Turgut F Ernst S-C Somfai GM Zoellin J Saad A. Implementation of a modified risk stratification score in cataract surgery at a Swiss public hospital. Clin Ophthalmol. (2025) 19:3307–16. 10.2147/OPTH.S544354 40949824 PMC12433236

[17] Spitznagel T Somfai G Turgut F Kang P Katz T Ernst S-C. Posterior capsule rupture and vitreous loss in cataract surgery: a retrospective analysis from a Swiss tertiary center. Clin Ophthalmol. (2026) 20:1–8. 10.2147/OPTH.S577514 PMC13055850 41952809

[18] Lin I-H Lee C-Y Chen J-T Chen Y-H Chung C-H Sun C-A. Predisposing factors for severe complications after cataract surgery: a nationwide population-based study. J Clin Med. (2021) 10(15):3336. 10.3390/jcm10153336 34362122 PMC8347944

[19] Danziger GS French DD Li Y Andrews CA Marwah S Rivera AS. Rates and associated risk factors for cataract surgery complications in academic medical centers. J Cataract Refract Surg. (2026) 52(3):223–30. 10.1097/j.jcrs.0000000000001822 41168667 PMC12928775

[20] Gedde SJ Feuer WJ Lim KS Barton K Goyal S Ahmed II. Treatment outcomes in the primary tube versus trabeculectomy study after 5 years of follow-up. Ophthalmology. (2022) 129(12):1344–56. 10.1016/j.ophtha.2022.07.003 35835337 PMC9691551

[21] Penzner J Sun X Wang YX Li N Issa M Lai H. Quantitative anterior segment SD-OCT assessment of the iridocorneal angle in Chinese patients with open-angle glaucoma or ocular hypertension: an observational study. Clin Ophthalmol. (2025) 19:4725–35. 10.2147/OPTH.S536846 41425738 PMC12717792

[22] Mastropasqua L Brescia L Oddone F Sacchi M Aloia R Totta M. Conjunctival thickness as a predictive imaging biomarker for the glaucoma filtration surgery outcome: an optical coherence tomography study. Clin Exp Ophthalmol. (2020) 48(9):1192–200. 10.1111/ceo.13857 32918375

[23] Sugihara K Ida C Ohtani H Murakami K Iida M Harano A. Geriatric assessment and risk prediction of severe complications in glaucoma surgery. J Glaucoma. (2026) 35:536–44. 10.1097/IJG.0000000000002742 42384722

[24] Groothoff J Rhead J Miller I De la Osa N Perry J Duy W. The relationship of frailty with surgical and laser treatment for patients with glaucoma. OPTH. (2025) 19:1455–65. 10.2147/OPTH.S514689 PMC12052001 40330005

[25] Halawa OA Kang J Parikh AA Oh G Glynn RJ Friedman DS. Relationship between claims-based frailty index and eye care utilization among medicare beneficiaries with glaucoma. Ophthalmology. (2023) 130(6):646–54. 10.1016/j.ophtha.2023.01.015 36731798 PMC10614114

[26] Funk IT Strelow BA Klifto MR Knight O’RJ Van Buren E Lin F-C. The relationship of travel distance to postoperative follow-up care on glaucoma surgery outcomes. J Glaucoma. (2020) 29(11):1056–64. 10.1097/IJG.0000000000001609 32694285 PMC7658019

[27] Shaheen A Medeiros FA Swaminathan SS. Association between greater social vulnerability and delayed glaucoma surgery. Am J Ophthalmol. (2024) 268:123–35. 10.1016/j.ajo.2024.07.019 39089357 PMC11606798

[28] Ren Y Adiyeke E Guan Z Hu Z Loftus TJ Shickel B. Mysurgeryrisk model predictions of postoperative complications and mortality. JAMA Surg. (2026) 161(6):619. 10.1001/jamasurg.2026.1112 42054034 PMC13130070

[29] Mahajan A Esper S Oo TH McKibben J Garver M Artman J. Development and validation of a machine learning model to identify patients before surgery at high risk for postoperative adverse events. JAMA Netw Open. (2023) 6(7):e2322285. 10.1001/jamanetworkopen.2023.22285 37418262 PMC10329211

[30] Esper SA Holder-Murray J Meister K Sylvia Lin H-H Bauer AK Artman J. Reducing unnecessary preoperative testing through a comprehensive EMR based digital algorithm. EBioMedicine. (2025) 111:105509. 10.1016/j.ebiom.2024.105509 39719798 PMC11731619

[31] Sim EY Tan DJA Abdullah HR. The use of computerized physician order entry with clinical decision support reduces practice variance in ordering preoperative investigations: a retrospective cohort study. Int J Med Inform. (2017) 108:29–35. 10.1016/j.ijmedinf.2017.09.015 29132628

[32] Borab ZM Lanni MA Tecce MG Pannucci CJ Fischer JP. Use of computerized clinical decision support systems to prevent venous thromboembolism in surgical patients: a systematic review and meta-analysis. JAMA Surg. (2017) 152(7):638. 10.1001/jamasurg.2017.0131 28297002 PMC5831455

[33] FDA. Clinical Decision Support Software—Guidance for Industry and Food and Drug Administration Staff. (2026).

[34] Xu M Chen W Li Z Xing Y Shang Y Yang C. Cumulative social determinants of health and frailty risk in older adults. BMC Geriatr. (2025) 25(1):895. 10.1186/s12877-025-06582-5 41225365 PMC12613642

[35] Yan Z Xu Y Du X. Social determinants of health, biological aging, and their impact on frailty risk: mediation and interaction effects analysis. Geriatr Gerontol Int. (2026) 26(1):e70331. 10.1111/ggi.70331 41537522

